# A Multicenter Survival Analysis of Adults Type B‐Acute Lymphoblastic Leukemia in Ecuador. A Retrospective Study of the Real World

**DOI:** 10.1002/cam4.71625

**Published:** 2026-02-26

**Authors:** Brenner Sabando, Jairo Quinonez, Andrés Orquera, Danilo Navarrete, Jhoanna Ramírez, Lorena Sanchez, Teodoro Chisesi, Jorge Oliveros, María A. Pacheco, María C. Trujillo, Carlos Plaza, Rafael Lochamin, D. Jimmy Martin

**Affiliations:** ^1^ IRHED Medical Specialties Center Guayaquil Ecuador; ^2^ Hematology Service Carlos Andrade Marín Hospital Quito Ecuador; ^3^ Hemato‐Oncology and Medicine Transfusion Department Solca Cancer Institute Manabí Ecuador; ^4^ Hematology Service Teodoro Maldonado Carbo Hospital Guayaquil Ecuador; ^5^ Department of Hematology Abel Gilbert Ponton Hospital Guayaquil Ecuador; ^6^ Angela Serra Association for Cancer Research Lecce Italy; ^7^ Hematology Service Luis Vernaza Hospital Guayaquil Ecuador; ^8^ Hemato‐Oncology and Bone Marrow Transplant Unit Solca Cancer Institute Cuenca Ecuador; ^9^ Department of Hematology Eugenio Espejo Hospital Quito Ecuador; ^10^ Hemato‐Oncology and Medicine Transfusion Department Solca Cancer Institute Guayaquil Ecuador

**Keywords:** acute lymphoblastic leukemia, adults, mortality, survival

## Abstract

**Objective:**

To describe clinical characteristics, treatment outcomes, and overall survival (OS) of Ecuadorian adults with B‐cell acute lymphoblastic leukemia (B‐ALL).

**Methods:**

A retrospective multicenter cohort study was conducted in patients > 15 years diagnosed with B‐ALL between 2015 and 2022 across eight tertiary centers in Ecuador. Medical records of 734 acute lymphoblastic leukemia cases classified by the 2016 WHO criteria were reviewed; 653 B‐ALL patients were included. Multiple frontline chemotherapy regimens were used, and Philadelphia‐positive (Ph+) cases were included.

**Results:**

Among 653 patients, 50.4% were male, with a median age of 31 years (interquartile range, 20–47). Most (93%) received chemotherapy, achieving a complete remission (CR) rate of 53.3% (311/583) and minimal residual disease (MRD) negativity in 49.1% (211/430). Relapse occurred in 62.4% (194/310), and 16 of 164 underwent allogeneic hematopoietic stem cell transplantation (HSCT). Treatment‐related mortality was 31.5%, higher in pediatric‐inspired protocols (34.4% vs. 21.3%, *p* < 0.001). Patients < 30 years showed better CR (58.3%, *p* = 0.026) and longer survival (median OS 15 vs. 8 months, *p* < 0.001). Median OS for the cohort was 11 months, with a 5‐year OS of 22.4%. High‐risk patients had inferior OS (11 vs. 14 months, *p* = 0.036), while no difference was observed between Ph + and Ph– (12 vs. 11 months, *p* = 0.319).

**Conclusion:**

Adult Ecuadorian patients with B‐ALL show lower survival than international cohorts, mainly due to high treatment‐related mortality and limited transplantation access. Age was independently associated with response and OS.

## Graphical abstract

Analysis of adults type b‐acute lymphoblastic leukemia in ecuador. This demonstrates inferior response and survival outcomes for b‐all in Ecuador and can serve as a reference to Latin America countries due to geographic, racial and socioeconomic similarities.

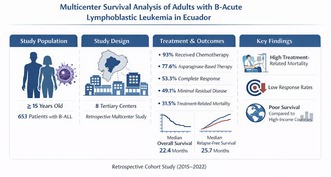

## 1 Introduction

Acute lymphoblastic leukemia (ALL) is a malignant hematologic disorder and the most common cancer in children [1, 2], as well as the second most frequent acute leukemia in adults. Its pathogenesis involves complex genetic and chromosomal abnormalities [3].

ALL shows a bimodal distribution, with about 80% of cases in children and 20% in adults [4]. Globally, incidence has increased from 1990 to 2017 [5]. In the United States, approximately 6000 individuals are diagnosed annually, 40% aged 15 years or older [6, 7]. The disease is less common in Black and Asian populations but more frequent among Hispanics, who also show a higher incidence of B‐cell ALL and Philadelphia chromosome–like subtypes. Young Hispanics are more likely to develop ALL than non‐Hispanics [8, 9, 10], and their age‐adjusted incidence rates remain significantly higher [11]. Although 5‐year overall survival for adults has improved in the U.S. and Western Europe, young Hispanic adults still show slightly lower survival than white Americans [12, 13, 14, 15].

In Latin America, adults ‐ ALL outcomes remain lower than those reported in high‐income countries, despite efforts to adapt international protocols. Prospective studies using modified CALGB 10403 and BFM (LLA 15–30) regimens achieved survival rates comparable to global benchmarks [16, 17], while most retrospective analyses still report inferior results [18, 19, 20, 21, 22, 23, 24, 25, 26].

Treatment has undergone changes, modified pediatric regimens improve outcomes in adolescent and young adult patients, while dose‐adjusted chemotherapy and novel targeted or immunotherapies benefit older adults [27, 28, 29, 30, 31, 32, 33, 34, 35, 36, 37].

In Ecuador, there are no multicenter studies for adults‐ALL; thus, our study aims to describe clinical and demographic characteristics, as well as response outcomes and OS of adults Ecuadorian diagnosed with B‐ALL in the period from 2015 to 2022.

## 2 Materials and Methods

### 2.1 Study Design and Data Collection

We conducted a retrospective cohort multicenter study of patients age 15 years and older with B‐ALL from January 2015 to December 2022 in 8 reference centers in Ecuador. Pregnant patients were not included. This study was reviewed and approved by the Human Investigation Committee (IRB) of the Technical University of Manabí (CEISH‐UTM‐EXT_23–01‐5_BESV), and all participant centers approved this study. Due to the methodology design, our study was classified as minimal risk in humans, and the informed consent form was waived by IRB.

Diagnosis of B‐ALL was based on the World Health Organization (WHO) 2008 criteria [38]. For all patients, complete blood count at diagnosis was recorded, and bone marrow aspiration, as well as bone marrow biopsy, were performed. Lineage of ALL was determined by immunohistochemistry and/or flow cytometry (FC).

The data registry included karyotype by conventional technique, BCR:ABL fusion determined by fluorescence in situ hybridization (FISH) or molecular biology (polymerase chain reaction), KTM2A rearrangement detected by FISH. Central nervous system (CNS) infiltration was evaluated through cerebrospinal fluid (CSF) analysis. Fifty patients were assessed by conventional cytology, and the remainder by FC. CNS involvement was defined as the presence of lymphoblasts on cytology or ≥ 5 leukocytes/μL and/or lymphoblasts detected by FC [39]. Stratification of risk was defined as (1) high risk: age ≥ 35 years and/or leukocytes ≥ 30,000/mm3 and/or CNS infiltration at baseline; and (2) standard risk if none of these factors were present [40].

### 2.2 Treatment and Response to Treatment

Treatment protocols recorded were classified as pediatric‐inspired protocols and adult‐designed protocols. Six different pediatric protocols were recorded: BFM [41], PETHEMA [42], CALGB 8811 [36], GATLA [19], Total XV [43], Failler [32]. Asparaginase‐based protocols were implemented using non‐pegylated, non‐Erwinia asparaginase; pegylated asparaginase is not available in Ecuador.

Adult protocols recorded were HyperCVAD/MTX‐ARAC [44], Rituximab + HyperCVAD [45], Flag‐Ida [46], and augmented HyperCVAD [47]. Rituximab use was restricted to patients treated with Hyper‐CVAD plus rituximab. Protocols that did not fulfill curative purpose were classified as palliative. Seventeen individuals declined or abandoned treatment. In addition, hematopoietic stem cell transplantation (HSCT) was registered.

Philadelphia chromosome–positive (Ph+) patients were treated with tyrosine kinase inhibitors (TKIs) in addition to chemotherapy, predominantly imatinib as the first‐line agent. One patient required a switch to dasatinib due to inadequate response. TKI administration was recorded as part of frontline therapy for all eligible Ph+ subjects.

Regarding novel therapies, availability in Ecuador remains limited. Blinatumomab is the only new agent currently accessible; however, no patient in this cohort received it.

Antimicrobial prophylaxis practices were not standardized across centers; however, most institutions followed NCCN‐based regimens, typically trimethoprim–sulfamethoxazole 160/800 mg on Mondays, Wednesdays, and Fridays, acyclovir 800 mg daily, and fluconazole 150 mg daily for non‐neutropenic patients.

Response was defined by: (1) post‐induction complete response (CR), (2) post‐induction minimal residual disease (MRD), (3) Treatment‐related mortality (TRM), (4) relapse, (5) Relapse‐free survival (RFS), (6) 5‐year OS, (7) median OS, (8) Event‐free survival (EFS). CR was evaluated according to Cheson criteria, defined as absence of extramedullary disease, absence of peripheral blasts, bone marrow blasts < 5%, neutrophil count ≥ 1.5 × 109/L, and platelets ≥ 100 × 109/L, any different result was classified as non‐response (NR) [48]. MRD was evaluated by FC, defined as a value < 0.01, which translates into 1 cell per 10,000 cells among all mononuclear cells in a bone marrow sample [49]. TRM was defined as the proportion of patients undergoing chemotherapy who die as a direct consequence of treatment‐associated complications or toxicities, independent of disease progression or other unrelated causes.

Relapse was defined as CR patients who were subsequently detected ≥ 5% blasts in the bone marrow, evidence of extramedullary disease, or the presence of blasts in the peripheral blood, based on the consensus of the European Working Group for Adult ALL (EWALL) [50]. RFS was defined as the time from the date of CR until the patient's relapse, death or last follow‐up, whichever comes first. The 5‐year OS, as well as the median OS, were defined as the time from diagnosis until the patient's death, or last follow‐up. EFS was defined as the time from diagnosis until the patient's relapse, death or last follow‐up, whichever comes first.

### 2.3 Age Groups Definition

Subjects were classified into 3 age groups, based on the AYA classification. These groups were defined as 15–39 years, 40–64 years, and ≥ 65 years or older.

### 2.4 Statistical Analysis

Study variables were described as means or medians, depending on their distribution, and frequencies or percentages were used for categorical variables. Pairwise comparisons between subject groups were determined by Chi‐square methods, or Fisher's exact test for parametric or nonparametric distributions, respectively. Bivariate correlations were performed using Pearson's methods for variables with a normal distribution, and Spearman's Rho for those variables that did not exhibit normal distribution. ROC (Receiver operating characteristic) curve was applied to estimate the cutoff values of age associated response and OS outcomes and was compared to Akaike information criterion (cAIC) to find most accurate value [51, 52].

For survival analysis, 5‐year OS and median OS were determined by Kaplan–Meier statistic and variables compared by log rank or Tarone‐Ware methods. Factors associated with outcome responses (CR, MRD, and TRM) were calculated through logistic regression. Variables were first evaluated in univariable analyses for their association with treatment response and survival outcomes. Factors with statistically significant associations were subsequently included in the multivariable logistic regression and Cox proportional hazards models. Relapse was measured using cumulative incidence function with death as a competitor, and the Fine‐Gray model was performed to estimate risk factors significantly related to relapse risk.

Statistical analyses were performed using SPSS “Statistics for Windows, Version 25.0 (2015; IBM) and R software package version v 4.4.2 (R Foundation for Statistical Computing; www.r‐project.org). The level of statistically significant accepted was 5% (*p* < 0.05).

## 3 Results

Medical records of 734 patients were reviewed. Eighty one subjects were excluded for the following reasons: 37 were classified as T‐lineage ALL, 4 as mixed lineage ALL and 40 were exclude due to lack of data. Six hundred and fifty three patients with B‐ALL were included. 50.4% were men. Median age at diagnosis was 31 years [interquartile range (IQR) 20–47], 409 (62.6%) patients were in the AYA group. 607 (93%) patients received chemotherapy, 471 (77.6%) received a pediatric inspired protocol (6 different regimens) and 136 (22.4%) received an adult protocol. 266 (44.8%) patients were classified in standard and 328 (55.2%) in high‐risk group. Normal karyotype was reported in 343/528 (65.1%). BCR::ABL1 fusion was detected in 61/490 tested individuals (12.4%), and 46 of these patients received tyrosine kinase inhibitor therapy. KMT2A rearrangements were recorded in 15/120 (12.5%). CNS infiltration was identified in 122/559 (21.9%) patients. Clinical and demographic characteristics of the cohort are summarized in Table 1.

**TABLE 1:** Baseline characteristics of B‐ALL patients at diagnosis.

Variable		*n*	(%)
Median age	31 years (IQ 20–47); range (15–87)		
AYA classification	15–39 years	409	(62,6)
	40–64 years	194	(29,7)
	65 years or older	50	(7,7)
Age cutoff value^a^	≥ 30 years	342	(52.4)
	< 30 years	311	(47.6)
Sex	Female	324	(49,6)
	Male	329	(50,4)
BCR::ABL fusion	Negative	490	(100)
	Positive	429	(87,6)
		61	(12,4)
Number of leukocytes at diagnosis	< 30.000	653	(100)
	≥ 30.000–< 100.000	384	(58,8)
	≥ 100,000	183	(28)
		86	(13,2)
KTM2A rearrangement	Negative	120	(100)
	Positive	105	(87,5)
		15	(12,5)
CNS infiltration	Negative	559	(100)
	Positive by flow cytometry Positive by cytology	437	(78,1)
		106	(19)
		16	(2,9)
Risk stratification	Standard	594	(100)
	High	266	(44.8)
		328	(55.2)
Karyotype	Normal	527	(100)
	Other clonal alterations	343	(65,1)
	Too few metaphases	67	(12,7) (9,3)
	Hyperdiploid	49	(7)
	Low hypodiploid	37	(2,8)
	Complex	15	(3)
		16	
Treatment protocol	BFM	607	(100)
	HyperCVAD/MTX‐ARAC	309	(50,9)
	PETHEMA LAL 2011	130	(21,4)
	CALGB 8811	63	(10,4)
	Others^b^	46	(7,6)
		59	(9,7)
Pediatric protocols	Pediatric protocols	607	(100)
	Adult protocols	471	(77,6)
		136	(22,4)

*Note:* Asparaginase protocols: BFM, PETHEMA, CALGB 881, GATLA, Total XV, Failler. Adult protocols: HyperCVAD/MTX/ARAC, Rituximab + HyperCVAD, Flag‐Ida, augmented HyperCVAD.

Abbreviations: AYA, adolescent and young adult; CNS, Central Nervous System; IQ, Interquartile Range.

^a^ Best age cutoff value to response and survival outcomes in our cohort determined by Receiver operating characteristic (ROC) curve and Akaike information criterion (cAIC).

^b^ This includes Total XV, Flag‐Ida, Failler, Rituximab + Hyper‐CVAD, augmented Hyper‐CVAD.

### 3.1 Response to Treatment

583/607 subjects received chemotherapy were evaluated. CR was achieved in 311 (53.3%) patients. MRD was observed in 211/430 (49.1%).

### 3.2 Mortality

The overall treatment‐related mortality (TRM) rate was 31.5%, with 23.3% occurring during induction and 8.2% during consolidation. Pediatric‐inspired protocols were associated with a higher TRM compared to adult regimens (34.4% vs. 21.3%, *p* < 0.001); however, this effect was largely driven by age. Among patients aged ≥ 30 years, the use of pediatric protocols significantly increased TRM [41.9% vs. 26.8% in younger patients; OR 3.2 (95% CI 1.9–5.1); *p* < 0.001].

In the multivariable logistic regression model, patients younger than 30 years showed a borderline significant association with higher CR rates (OR 1.7; 95% CI 1.0–2.9; *p* = 0.051). Pediatric‐inspired protocols and absence of CNS infiltration were independently associated with achieving MRD negativity (OR 5.8; 95% CI 2.3–11.0; *p* < 0.001; and OR 2.7; 95% CI 1.1–6.8; *p* = 0.028, respectively). Patients aged < 30 years also had significantly lower TRM (OR 0.3; 95% CI 0.1–0.5; *p* < 0.001). In contrast, a normal karyotype was associated with higher TRM compared with abnormal karyotype (OR 2.7; 95% CI 1.4–5.5; *p* = 0.003). Factors associated with response outcomes are summarized in Table 2.

**TABLE 2:** Multivariate regression logistic model of factors associated to outcomes.

Factors	Outcomes		
	CR OR [(95% CI) *p*]	Negative MRD OR [(95% CI) *p*]	TRM rate^a^ OR [(95% CI) *p*]
Male vs. female	50.8 vs. 51.6 OR 1.1[(CI 0.6–1.8) *p* = 0.877]	52.8 vs. 45.8 OR 0.8[(CI 0.5–1.3) *p* = 0.346]	35 vs. 28.8 OR 1.4[(CI 0.7–2.5) *p* = 0.252]
Phi+ vs. Phi−	56.6 vs. 51.3 OR 1.1[(CI 0.5–2.5) *p* = 0.847]	53.2 vs. 36.4 OR 0.91[(CI 0.4–2.1) *p* = 0.817]	28.3 vs. 30.8 OR 0.7[(CI 0.3–1.8) *p* = 0.578]
Pediatric vs. adults protocols	50.7 vs. 51.7 OR 1.6[(CI 0.7–3.4) *p* = 0.173]	57.5 vs. 20.4 OR 54.8[(CI 2.3–11) *p* < 0.001]	34.4 vs. 21.3 OR 1.1[(CI 0.5–2.8) *p* = 0.758]
< 30 vs. > = 30 years old	57.5 vs. 45.4 OR 1.7[(CI 1.0–2.9) *p* = 0.051]	55.9 vs. 41.5 OR 1.6[(CI 0.9–2.6) *p* = 0.080]	25 vs. 34 OR 0.3[(CI 0.1–0.5) *p* < 0.001]
No CNS infiltration vs. CNS infiltration	54.1 vs. 54.2 OR 1.7[(CI 0.7–4.2) *p* = 0.204]	53.1 vs. 40.9 OR 2.7[(CI 1.1–6.8) *p* = 0.028]	28.8 vs. 30.3 OR 0.6[(CI 0.2–2.3) *p* = 0.535]
Standard risk vs. high risk group	53.9 vs. 52.2 OR 2.6[(CI 0.6–11) *p* = 0.174]	51.5 vs. 49.1 OR 1.6[(CI 0.4–5.6) *p* = 0.480]	32 vs. 29 OR 0.9[(CI 0.1–4.7) *p* = 0.947]
Under vs. over 100 mm3 leukocytes	52.7 vs. 41.2 OR 2[(CI 0.8–4.7) *p* = 0.094]	50.5 vs. 40 OR 1.3[(CI 0.5–3.2) *p* = 0.493]	31.6 vs. 33.7 OR 0.9[(CI 0.3–2.7) *p* = 0.884]
Normal vs. abnormal karyotype	72.8 vs. 70.7 OR 1[(CI 0.5–1.9) *p* = 0.958]	55.7 vs. 42.4 OR 1.5[(CI 0.8–2.7) *p* = 0.133]	55.3 vs. 39.8 OR 2.7[(CI 1.4–5.5) *p* = 0.003]

*Note:* Multivariate logistic regression model of factors associated with Complete Response (CR), Minimal Residual Disease (MRD), and Treatment‐Related Mortality (TRM). Percentages are calculated within each evaluated factor. For each variable, the second category listed in the “Factor” column is used as the reference in the logistic regression model.

^a^ TRM includes mortality occurring during induction and consolidation.

### 3.3 Relapse

**FIGURE 1:**
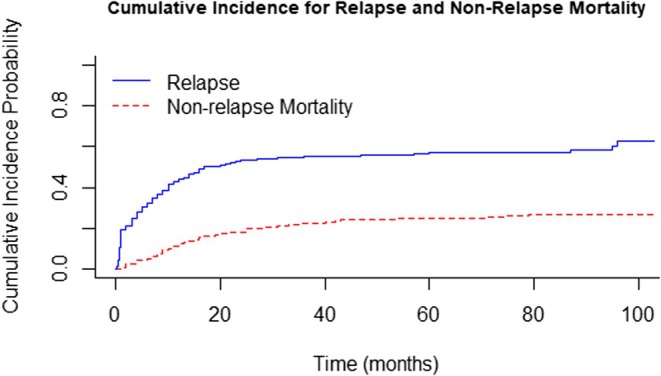
Cumulative incidence curves depicting relapse and non‐relapse mortality (NRM) using a competing‐risk framework.

Median and 5‐year RFS was 14 months (95% CI 11–19) and 25.7%, respectively. Cumulative incidence for relapse (CIR), considering death as a competitor event, was 48.1% (95% CI 41.8–55.1), while the non‐relapse mortality (NRM) throughout the follow‐up was 43.5% (95% CI 24.6–62.5), (Figure 1). Leukocytes count was associated to CIR, 45.3%, 45.7%, 67.9% (< 30,000/mm^3^ vs. 30,000–< 100,000/mm^3^ vs. > =100,000/mm^3^, *p* = 0.013).

### 3.4 Survival Analysis

**FIGURE 2:**
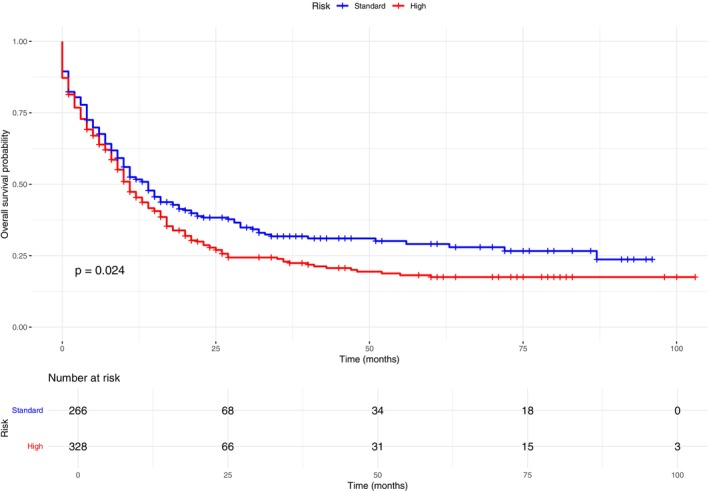
Kaplan–Meier overall survival (OS) according to risk stratification: Standard‐risk (blue) versus high‐risk (red) groups.

Median OS for total cohort was 11 months (95% CI 9.4–12.5), with a 5‐year OS of 22.4%. Median and 5‐year EFS for total cohort were 10 months (95% CI 8.6–11.4) and 18%, respectively. OS univariate analysis showed statistically significant differences for: high risk group was 11 months (95% CI 9.1–12.8) versus 14 months (95% CI 11–17) standard group (Figure 2). CR was 21 months (95% CI: 15.9–26.1) vs. 10 months (95% CI: 8.4–11.5) refractory patients. Negative MRD was 34 (95% CI 12.3–53.6) vs. 13 months (95% CI 10.6–15.3) positive MRD. Details of the survival analysis can be found in Table 3.

**TABLE 3:** OS and EFS univariate analysis of patients in chemotherapy according to group.

Factor	Median OS	5‐year OS	OS *p*	Median EFS [95%]	5‐year EFS	EFS *p*
AYA group			*p* < 0.001	14 [95% CI: 12–17]	14%	*p* = 0.005
< 40 years	14 [95% CI: 11.5–20]	26.4%		8 [95% CI: 5–10]	9%	
> = 40	7 [95% CI: 5.7–15]	12%				
< 30‐year old	8 [95% CI: 7.3–10.7]	14.2%	*p* < 0.001	9 [95% CI: 7.1–10.8]	15.8%	*p* = 0.036
No	15 [95% CI: 12.1–17.9]	30.5%		11 [95% CI: 8.5–13.4]	20.4%	
Yes						
Sex			*p* = 0.587	11 [95% CI: 8.7–13.2]	23.1%	*p* = 0.043
Male	11 [95% CI: 8.6–13.3]	23.1%		9 [95% CI: 7.2–10.8]	12.6%	
Female	11 [95% CI: 8.9–13.1]	20.1%				
Karyotype			*p* = 0.525	13 [95% CI: 10–16]	9%	*p* = 0.795
Normal	12 [95% CI: 10–14]	20%		11 [95% CI: 9–16]	15%	
Abnormal	11 [95% CI: 9–16]	25%				
Risk group	11 [95% CI: 9.1–12.8]	19.2%	*p* = 0.024	9 [95% CI: 7.3–10.6]	15.5%	*p* = 0.030
High	14 [95% CI: 11–17]	30.8%		13 [95% CI: 10.1–15.9]	23.7%	
Standard						
Complete Response			*p* < 0.001	10 [95% CI: 8.3–11.6]	9%	*p* < 0.001
No	10 [95% CI: 8.4–11.5]	11.9%		16 [95% CI: 12.7–19.3]	28.3%	
Yes	21 [95% CI: 15.9–26.1]	33.2%				
Negative MRD	13 [95% CI: 10.6–15.3]	11.9%	*p* < 0.001	11 [95% CI: 9.3–12.6]	10%	*p* < 0.001
No	34 [95% CI: 12.3–53.6]	44.5%		25 [95% CI: 12.2–37.7]	37.3%	
Yes						
Phi chromosome			*p* = 0.319	10 [95% CI: 8.4–11.5]	14.5%	*p* = 0.204
Negative	11 [95% CI: 9.0–12.9]	18.9%		11 [95% CI: 6.7–15.3]	33.8%	
Positive	12 [95% CI: 7.7–16.3]	30.2%				
KTM2A rearrangement			*p* = 0.457	10 [95% CI: 7.2–12.7]	19.9%	*p* = 0.683
Negative	10 [95% CI: 7.8–12.1]	24%		11 [95% CI: 7.6–14.4]	0%	
Positive	11 [95% CI: 7.3–14.6]	0%				
CNS infiltration at diagnosis			*p* = 0.254	11 [95% CI: 9–12.9]	21.2%	*p* = 0.383
No	12 [95% CI: 9.8–14.1]	25.3%		11 [95% CI: 8.2–13.8]	12.8%	
Yes	12 [95% CI: 9.3–14.7]	14.4%				
# of leukocytes/mm3 at debut			*p* = 0.026	11 [95% CI: 8.7–13.3]	20.1%	*p* = 0.030
< 30.000	11 [95% CI: 8.5–13.4]	24.2%		10 [95% CI: 7.7–12.2]	18.7%	
30.000	12 [95% CI: 9.5–14.4]	22.7%		7 [95% CI: 4.5–9.5]	8%	
< 100.000	9 [95% CI: 6.6–11.4]	9%				
> = 100.000						
Pediatric protocols	11 [95% CI: 7.5–14.5]	15%	*p* = 0.780	10 [95% CI: 7.3–12.7]	12.1%	*p* = 0.767
No	11 [95% CI: 9.3–12.7]	23%		10 [95% CI: 8.2–11.8]	19.2%	
Yes						

Abbreviations: CNS, Central Nervous System; EFS, Disease Free Survival; MRD, Minimal Residual Disease; OR, Overall survival.

### 3.5 Pediatric vs. Adult Protocols

**FIGURE 3:**
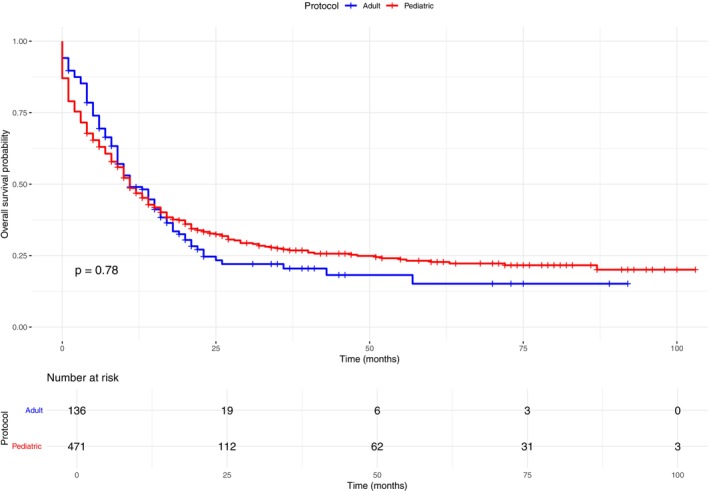
Kaplan–Meier overall survival (OS) by treatment protocol: Adult regimens (blue) versus pediatric‐inspired regimens (red).

**FIGURE 4:**
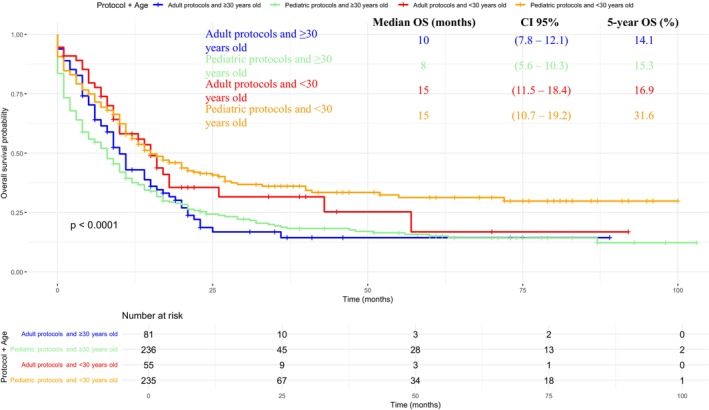
Kaplan–Meier overall survival (OS) comparing pediatric‐inspired and adult protocols within age subgroups (< 30 vs. ≥ 30 years).

**FIGURE 5:**
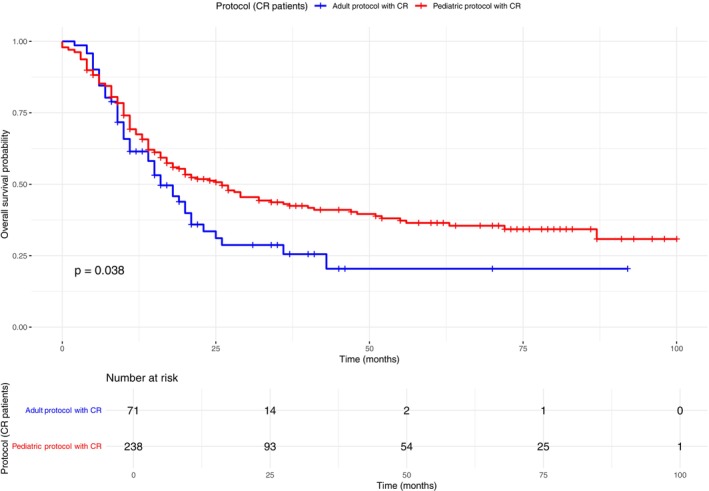
Kaplan–Meier overall survival (OS) among patients achieving complete remission (CR) and treated with pediatric‐inspired protocols, stratified by age (< 30 vs. ≥ 30 years).

Median OS was similar between pediatric‐inspired and adult protocols, 11 months median OS [95% CI 9.27–12.73 vs. 7.50–14.49; *p* = 0.780] (Figure 3). However, pediatric protocols were significantly associated with improved survival among patients younger than 30 years (Figure 4). This age‐dependent effect persisted even when restricting the analysis to patients who achieved CR. Among those receiving pediatric‐inspired therapy, < 30 years subjects median OS was 40 months (95% CI 21–NA) with a 5‐year OS of 44.9%, compared to 17 months (95% CI 13–29) and a 5‐year OS of 26.6% in ≥ 30 years old individuals (Figure 5).

### 3.6 Survival in Transplanted Patient

Of the 164 patients who belonged to the high‐risk group and achieved CR, 16 (9.76%) underwent allogeneic hematopoietic stem cell transplantation (HSCT), with a median OS of 27 months and a 5‐year OS of 74.3%. Twelve of the transplanted patients were alive at the term of the follow‐up period.

**FIGURE 6:**
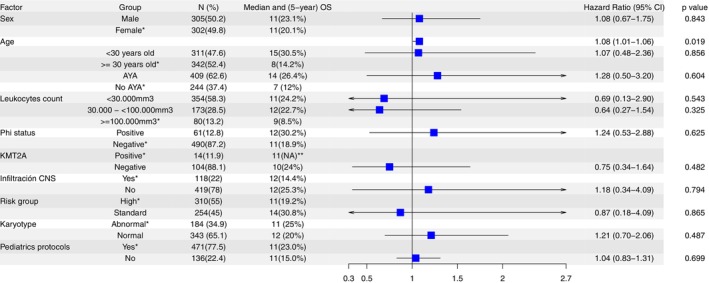
Multivariable forest plot illustrating hazard ratios for overall survival across clinical and demographic subgroups. *Indicates the reference category. **Indicates factors for which 5‐year overall survival (OS) was not reached.

In the multivariable Cox regression analysis restricted to patients receiving chemotherapy, age (as a continuous variable) was independently associated with worse overall survival [HR 1.08; 95% CI 1.01–1.06; *p* = 0.019]. The full multivariable survival model is presented in Figure 6.

## 4 Discussion

At the time of publication, this study represents the first national series describing clinical characteristics, treatment responses, and overall survival among adult patients with B‐ALL in Ecuador. The size and multicenter nature of this cohort provide a representative overview of the epidemiologic and clinical profile of Ecuadorian adults with this disease.

The demographic profile of our cohort is consistent with reports from other Latin American studies, where median age typically ranges from 29 to 34 years old [18, 19, 20, 21, 26]. This contrasts markedly with European cohorts, which report a substantially older median age of approximately 50 years [12, 13, 14]. A modest male predominance was also observed, aligning with patterns described in international series [5, 6, 13].

In terms of molecular features, the prevalence of Philadelphia chromosome–positive disease (12.4%) in our cohort falls at the lower end of the range reported in Latin America (12%–30%) [11, 18, 53] and below the rates typically described in Europe and the United States (20%–30%) [11, 54, 55]. Regarding conventional cytogenetics, clonal abnormalities were identified in 12.7% of evaluated patients, similar to other reports from the region (15%–30%) [16, 17, 18] but markedly lower than U.S., and European adult ALL cohorts (60%–80%) [44, 55].

KTM2A rearrangements were detected in 12.5%, consistent with the 5%–15% prevalence reported in adults [56, 57]. CNS infiltration was documented in 21.9% of patients, a higher proportion than that reported internationally (< 10%) [33, 35, 40]. Notably, the use of flow cytometry—which can detect CNS involvement in up to 28% of cases due to its higher sensitivity—likely contributed to this increased detection rate [58].

Response outcomes, including CR and MRD negativity, were lower than those reported in the U.S., European, and other Latin American cohorts [12, 17, 18, 59, 60]. Age was the most significant independent predictor of treatment response. Although pediatric‐inspired protocols yielded higher CR and MRD‐negative rates compared with adult regimens, this advantage was strongly mediated by age. TRM was also higher in our cohort—34.4% with pediatric protocols versus 21.3% with adult protocols—and exceeded rates described internationally, including other Latin American studies [16, 17, 18, 19, 61]. However, the use of pediatric protocols in subjects age 30 years or older significantly increases TRM.

Survival outcomes demonstrated significantly poorer results, with 5‐year OS and DFS well below those published in high‐income settings [6, 13, 60, 62]. However, these outcomes align with rates described in other retrospective studies from Latin America [18, 20, 21, 63]. Age was independently associated with both OS and DFS, and this effect extended to treatment protocols: pediatric‐inspired regimens were significantly associated with improved survival among younger patients. A comparison of our survival outcomes with international cohorts is presented in Table 4.

**TABLE 4:** Comparison of outcomes in adults acute lymphoblastic leukemia studies.

Study (year)	Countries	Design	*N*	Median age (range)	Phi+ (%)	CR (%)	MRD (%)^a^	IRM (%)	OS (%); years	DFS (%); years or months
Present	Ecuador	Retrospective, multicenter	653	31 (15–87)	12.4	53.3	49.1; < 0.01%	23.3	(22.4); 5 years	(25.7); 5 years
Kantarjian (2004) [60]	United States	Prospective, multicenter	288	40 (15–92)	17	92	Unavailable	5	(38); 5 years	(38); 5 years
Rowe (2005) [40]	United Kingdom and United States	Prospective, multicenter	1521	No reported (15–59)	19	91	Unavailable	4.7	(38); 5 years	(46); 5 years
Lennmyr (2019) [12]	Swedish	National Register	937	53 (18–95)	34	91	Unavailable	10	(65); 5 years^b^	NA
Ribera (2020) [35]	Spain	Prospective, multicenter	89	20 (15–29)	No included	95	77.9; < 0.1%	0	(74); 5 years	NA
Crespo‐Solis (2018) [18]	Mexico	Retrospective, multicenter	559	28 (14–81)	16.7	69.2	Unavailable	10.6	(22.1); 3 years	(16); months
Rangel‐Patiño (2023) [16]	Mexico and Guatemala	Prospective, multicenter	95	23 (14–49)	No included	83.2	75.4; < 0.1%	7.4	(72.1); 2 years	(68.4); 2 years
Puga (2014) [17]	Chile	Prospective, multicenter	68	20 (15–30)	No included	91	Unavailable	6	(61.8); 3 years	(67.5); 3 years
Jerez (2023) [61]	Chile	Retrospective, single institution	67	29 (15–86)	21	83.3	76; < 0.0001%	Unavailable	(69); 2 years	(59); 2 years
Ferrari (2023) [19]	Argentina	Retrospective multicenter	171	22 (15–40)	No included	91	67; < 0.1%	2	(62); 2 years	NA
Jaime‐Pérez (2016) [20]	Mexico	Retrospective, single institution	94	33 (18–85)	26.3	71.3	Unavailable	7.4	(23.4); 5 years	NA
da Silva Junior (2018) [62]	Brazil	Retrospective, single institution	59	35 (17–71)	33.8	76	Unavailable	17	(15.3); 6 years	NA
Wellington F. Silva (2022) [64]	Brazil	Retrospective, single institution	104	37.5 (15–79)	42.3	Unavailable	Unavailable	10.8	(42.8); 3 years	NA
Wellington F. Silva (2021) [53]	Brazil	Retrospective multicenter	123	42 (14–81)	100^c^	78.8	33.9; < 0.1	14.6	(25.5); 4 years	(25.5); 4 years
Rodriguez (2005) [59]	Peru	Prospective, single institution	231	18 (14–57)	Unavailable	79	Unavailable	Unavailable	(55); 3 years	(32); 3 years

Abbreviations: CR, Complete response; DFS, Disease‐free survival; IRM, Induction mortality rate; MRD, Minimal residual disease; OS, Overall survival.

^a^ Values represent the percentage of individuals achieving MRD negativity, followed by the sensitivity of the flow cytometry method used for MRD assessment.

^b^ Reported for patients aged from 18 to 49 years old.

^c^ A study evaluating Philadelphia‐positive B‐lymphoblastic leukemia subjects.

Multiple factors likely contributed to the suboptimal response and survival outcomes observed in our cohort, including limited access to timely healthcare, socioeconomic constraints, and delays in diagnosis and treatment. High treatment‐related mortality and restricted access to transplantation also play major roles. Adapting intensive protocols in less‐resourced settings remains challenging due to limited supportive care and inconsistent availability of essential medications. Nonetheless, prospective initiatives from Brazilian and Mexican groups demonstrate that meaningful improvements are achievable even with constrained resources [16, 64]. Genetic factors, such as GATA3‐associated polymorphisms prevalent in Latin America, may further contribute to the poor survival observed [65, 66]. These combined factors may help explain the poor survival rates observed in our cohort.

Despite limitations, primarily related to the retrospective design, the lack of uniform response assessments, and the heterogeneity of treatment approaches across centers, this study represents an important contribution to the understanding of adult ALL in Ecuador. It provides a foundation for the development of context‐appropriate treatment strategies and adapted protocols that may ultimately help narrow the outcome gap between our population and those reported in high‐income countries.

## 5 Conclusion

This study, the largest series of adult B‐ALL in Ecuador, reveals markedly lower outcomes driven by high treatment‐related mortality and limited transplantation access. Age remained a key prognostic factor. These findings offer a crucial regional reference and highlight the urgent need for adapted strategies to prevent widening survival disparities.

## Author Contributions

**Brenner Sabando:** conceptualization (lead), data curation (equal), formal analysis (equal), funding acquisition (lead), investigation (lead), methodology (lead), project administration (equal), resources (lead), software (equal), supervision (lead), validation (lead), visualization (lead), writing – original draft (lead), writing – review and editing (lead). **Jairo Quinonez:** conceptualization (lead), data curation (lead), formal analysis (lead), investigation (lead), methodology (lead), project administration (lead), software (lead), supervision (equal), validation (lead), visualization (lead), writing – original draft (lead), writing – review and editing (lead). **Andrés Orquera:** conceptualization (equal), formal analysis (equal), investigation (equal), methodology (equal), project administration (equal), supervision (equal), validation (equal), writing – original draft (equal), writing – review and editing (lead). **Danilo Navarrete:** investigation (equal), methodology (equal), project administration (supporting), writing – original draft (supporting). **Jhoanna Ramírez:** methodology (equal), project administration (supporting), writing – original draft (supporting), writing – review and editing (supporting). **Lorena Sanchez:** investigation (supporting). **Teodoro Chisesi:** conceptualization (equal), investigation (equal), methodology (equal), project administration (equal), supervision (equal), writing – original draft (equal). **Jorge Oliveros:** methodology (supporting), project administration (supporting), writing – original draft (equal). **María A. Pacheco:** investigation (equal), methodology (supporting), project administration (supporting), writing – original draft (supporting), writing – review and editing (equal). **María C. Trujillo:** investigation (supporting), methodology (supporting), project administration (supporting), writing – review and editing (supporting). **Carlos Plaza:** investigation (supporting). **D. Jimmy Martin:** methodology (supporting), writing – review and editing (supporting). **Rafael Lochamin:** conceptualization (equal), project administration (equal), visualization (equal).

## Funding

This study received funding from the Varifarma pharmaceutical company for design and execution.

## Ethics Statement

This study was approved and authorized by the Human Research Ethics Committee of the Technical University of Manabí.

## Consent

Our study was classified as posing minimal risk in humans, and informed consent to access patient's information was authorized by each of the participating hospitals.

## Conflicts of Interest

The authors declare no conflicts of interest.

## Data Availability

The dataset used for the current study is available from the corresponding author upon reasonable request.
